# Evidence for the use of spinal collars in stabilising spinal injuries in the pre-hospital setting in trauma patients: a systematic review

**DOI:** 10.1007/s00068-020-01576-x

**Published:** 2020-12-21

**Authors:** Katherine Hawkridge, Ikhlaaq Ahmed, Zubair Ahmed

**Affiliations:** 1 Neuroscience and Ophthalmology, Institute of Inflammation and Ageing, College of Medical and Dental Science, University of Birmingham, Birmingham, B15 2TT UK; 2 Cancer Research UK Clinical Trials Unit, University of Birmingham, Birmingham, B15 2TT UK; 3 Surgical Reconstruction and Microbiology Research Centre, National Institute for Health Research, Queen Elizabeth Hospital, Birmingham, B15 2TH UK

**Keywords:** Spinal injuries, Pre-hospital, Acute treatment, Trauma, Spinal collars, Trauma management

## Abstract

**Purpose:**

Spinal collars were introduced in 1967 into the management of spinal trauma care as it was thought that this technique of immobilisation would prevent any further neurological or spinal damage in high-risk patients. The aim of this systematic review was to determine whether the use of spinal collars in the pre-hospital trauma patient was recommended by published literature.

**Methods:**

A systematic search of the literature was conducted between 1990 and 2020, screening PubMed, Medline, Science Direct and Google Scholar. The consequent findings were then qualitatively synthesised with the aim of effectively evaluating the evidence to resolve the discrepancy between current practice and literature.

**Results:**

Of the nine eligible studies, six deemed that spinal collars should not be used in pre-hospital trauma patients with the remaining three reporting uncertainty if spinal collars were best practice. Our results suggest that there is a discrepancy between current guidance and practice in that although the guidelines recommend the use of spinal collars in the pre-hospital setting the majority of the studies were against the use of spinal collars. Importantly, none of the studies reported any benefits of spinal collars.

**Conclusion:**

Our study shows a disparity between current guidelines and the published literature and warrants further direct research to obtain a more comprehensive view of the use of spinal collars in a pre-hospital setting.

## Introduction

Spinal Injury (SI) affects around 1000 people every year in the UK and an estimated 102,000 to 1.2 M new cases worldwide, with survivors experiencing life-long loss of function and reduced mobility [1]. The use of spinal collars to immobilise the spine in the pre-hospital setting for suspected spinal injury is recommended by the National Institute for Health and Care Excellence (NICE) and the Joint Royal Colleges Ambulance Liaison Committee (JRCALC). Current guidelines are based on the premise that immobilisation will prevent further neurological damage in patients with SI [2]. There are many different protocols to support the decision about immobilisation and these vary regionally, nationally and internationally [3–7]. However, the use of such spinal collars is currently under debate [3]. In recent years, several studies have questioned the efficacy and effectiveness of spinal collars for SI patients. A Cochrane review in 2001 discovered that of the 4453 relevant articles, no articles reported a randomised controlled trial (RCT) to support the use of spinal collars [8]. An RCT study of this nature poses obvious problems in being carried out which includes questions as to whether such a study would be approved ethically. A systematic review in 2005 presented that although pre-hospital spinal collars provided some benefits, adverse effects of such collars also reported pain and discomfort [9].

Current pre-hospital guidelines in the UK are led by the JRCALC, NICE and local ambulance services guidelines. JRCALC states that all patients with the likelihood of SI should be immobilised at the earliest time possible, while initial assessments are undertaken. It also states that if immobilisation is indicated then the whole spine must be immobilised; where acceptable, methods of immobilisation are a collar, head blocks and spinal support (https://www.jrcalc.org.uk/guidelines/). On the other hand, NICE states that spinal collars should be used to immobilise the spine to prevent any movement and help assist in avoiding any secondary SI [10]. However, over the last 5 years, it has been left to the discretion of the pre-hospital clinician as to whether spinal collars are a necessity. Along with this, there have emerged many different protocols that pre-hospital clinicians can use to support their decision about immobilisation [4, 5]. Indeed, a study amongst German paramedics found that most Paramedics were certain about their competence to assess patients in the pre-hospital environment and whether they required spinal immobilisation [6]. Nevertheless, this can leave certain pre-hospital clinicians with a level of confusion on what is the best evidence-based practice to give their patient to conform to a gold standard of care.

The aim of this article is to review the literature relevant to the use of spinal collars in the pre-hospital setting. This is to evaluate, analyse and contrast elements and draw a conclusion on the effectiveness of spinal collars in stabilising SIs in the pre-hospital setting in trauma patients. An additional aim will be to resolve the current confusion around the topic area for pre-hospital clinicians. This will result in giving all trauma patients a consistent level of care, founded on evidence-based practice.

## Methods

### Literature search

We used the search strategies recommended for the Preferred Reporting Items for Systematic reviews and Meta-Analysis (PRISMA) statement [11] and the Cochrane Handbook for Systematic Reviews of Interventions [12] to ensure methodological accuracy. Titles, abtracts, key words, and free text were searched using combinations of the following key words: “Pre-hospital immobilisation”, OR “Trauma patients spinal injury management”, OR “Spinal collars effectiveness”. We searched PubMed, Medline, Science Direct and Google Scholar from 1990 to 5th May 2020 for all studies that reported data on the use of spinal collars in the pre-hospital setting.

### Inclusion and exclusion criteria

The studies to be included were screened separately using the following criteria (Table 1): (1) articles were written in English, peer-reviewed, conference articles and that the data was from a pre-hospital setting; (2) studies in adults aged between 16 and 99 years and inclusive of all mechanism of traumatic injuries. Exclusion criteria: (1) patients with old spinal injuries; (2) children under 16 years old; (3) patients who are known to be immunocompromised and not deemed as “healthy patients”; and (4) animal studies, in vitro, simulations in virtual reality or by computer.

**Table 1:** Inclusion and exclusion criteria

Inclusion criteria	Exclusion of criteria
Adults aged 16–99 years	Patients who have old spinal injuries
All mechanisms of traumatic injuries	Children under 16 years old
Papers published since 1990	Patients who are known to be immunocompromised and not deemed as “healthy patients”
Written in English only	Animal studies, in vitro, simulations in virtual reality or by computer

### Data collection process

Two reviewers (K.H. and Z.A.) independently conducted the literature search based on the title and abstract and undertook the full-text review. In case of disagreement, this was resolved through discussion. Relevant articles had to meet the inclusion/exclusion criteria, as stated in Table 1 above. Records were imported into Reference Manager, duplicates removed, titles and abstracts were screened manually, and full texts of potential studies were retrieved.

### Data extraction and synthesis

The following data is extracted: (1) study characteristics; (2) patient characteristics (number of patients and demographics); (3) aims of study; (4) main findings relevant to the use of spinal collars.

### Quality assessment and statistical analysis

The modified Newcastle–Ottawa scale (NOS) (http://www.ohri.ca/programs/ clinical_epidemiology/oxford.asp) was used for assessing the quality and risk of bias of the included studies. One reviewer (ZA) assessed the risk of bias of each study using this scale and high, moderate and low risk of bias were defined as NOS < 4, between 4 and 6, and > 6, respectively, as in the original NOS. The risk of publication bias was further assessed using Egger’s test. A *p* value of less than 0.1 for Egger’s test was considered statistically significant.

### Statistical analysis

Odds ratio (OR) was calculated with a Fixed effect analysis model calculated using RevMan 5.3 (Nordic Cochrane Centre, Copenhagen, Denmark). We analysed the OR of each of the predictive factors using the generic method of the inverse of the variance to combine these data, some studies did not explicitly report them, which we calculated using the OR calculator of Review manager. Heterogeneity was assessed by calculating Chi-square (*I*^2^), with a high heterogeneity of the studies included in the analysis being above 60%.

## Results

### Study selection

**Fig. 1:**
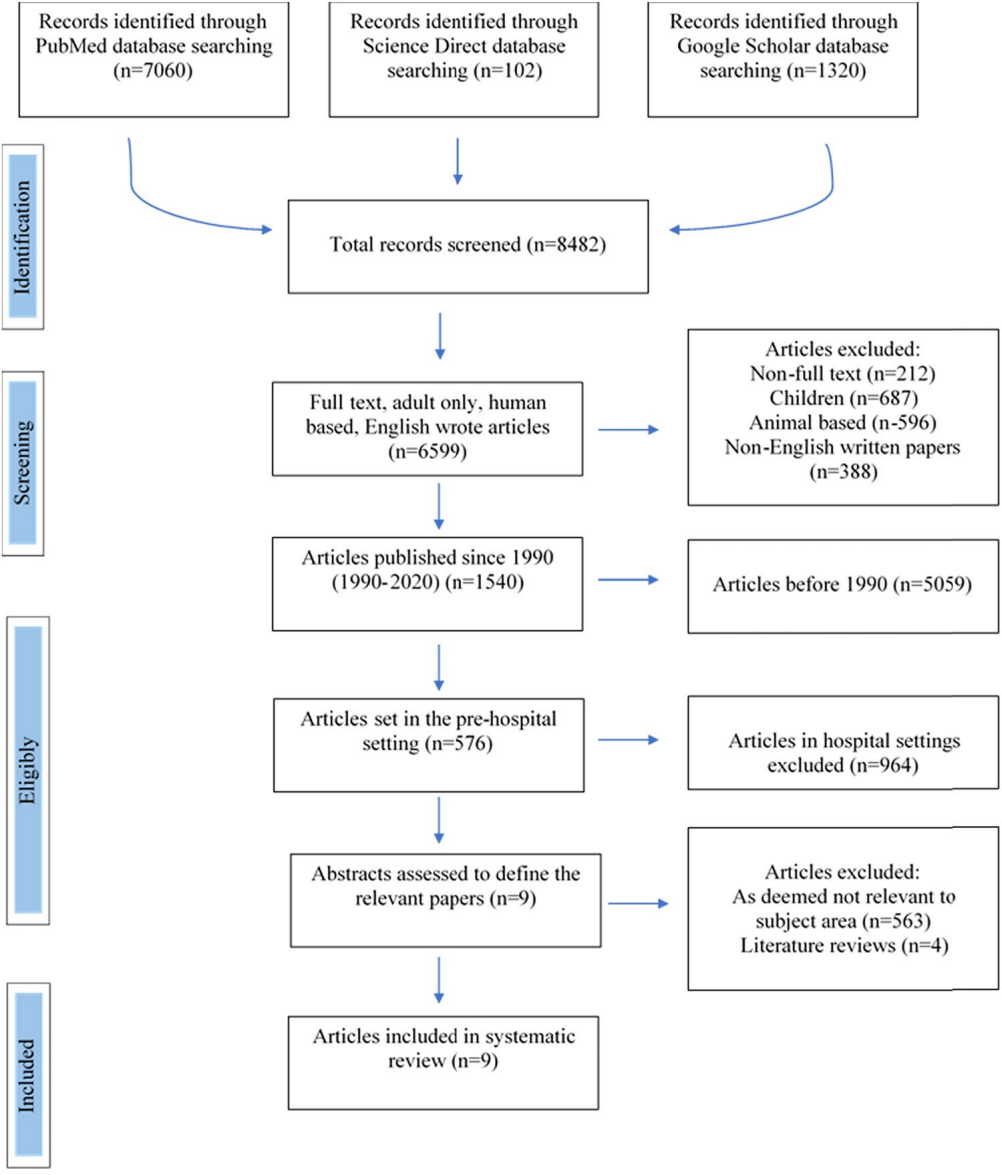
PRISMA flow chart of the screening process

After conducting the systematic search of the information following the PRISMA strategy, 8482 records were found in PubMed, Science Direct and Google Scholar (Fig. 1). From these, we excluded 212 non-full-text articles, 687 that included children, 596 that were animal-based studies and 388 articles which were not originally written in English. We also excluded 5059 records that were published before 1990. Of the 1540 remaining records, 576 were included as they were set in the pre-hospital environment. The remaining articles had their titles and abstracts read and 567 were excluded as they were not relevant to the key subject area, including 4 records that were literature reviews. This left a total of 9 records which were then critically analysed.

### Study characteristics

From the studies included, all nine were retrospective studies; two were qualitative in design, whilst seven were quantitative. The study locations were varied with the majority being undertaken in the United States of America (USA) (*n* = 4), Netherlands (*n* = 1), Israel (*n* = 1), Asia (*n* = 1), unknown location (*n* = 1) and multiples locations (*n* = 1). This assists the systematic review by reducing the culture bias. The overview of the reviewed articles is given in Table 2.

**Table 2:** Characteristics of the included studies

Study	Study location	Type	Patients	GRADE quality	Main findings relevant to pre-hospital use of collars
Underbrink et al. [21]	Rocky Mountain	4-year retrospective study	5063	High	No differences in neurologic deficit or patient disposition in the older adult patient with cervical spine trauma despite changes in spinal restriction protocols and resulting differences in immobilization devices
Oosterwold et al. [19]	Netherlands	Retrospective observational study	1082	Moderate	Consensus among EMS staff on how to interpret the criterion ‘distracting injury’ was lacking. Adverse effects of spinal immobilisation were incompletely documented in pre-hospital care reports. To provide validated information on potential symptoms of SCI, a uniform EMS scoring system for motoric assessment should be developed
Vanderlan et al. [22]	Louisiana State, USA	Retrospective observational study	199	Moderate	Cervical spine immobilisation was associated with an increased risk of death (*p* < 0.02, Odds ratio 2.77, 95% CI 1.18–6.49)
Haut et al. [16]	USA	Retrospective observational study	45,284	High	Pre-hospital spine immobilisation was associated with higher mortality in penetrating trauma and should not be routinely used in every patient with penetrating trauma
Brown et al. [14]	New York, USA	Retrospective observational study	75,567	High	Documented benefits of pre-hospital spinal immobilisation in patients with torso gunshot wounds remains unproven, despite the potential to interfere with emergent care in this patient population
Hauswalk et al. [15]	Malaysia and New Mexico	Retrospective observational study	454	Moderate	Out-of-hospital immobilisation has little or no effect on neurologic outcome in patients with blunt spinal injuries
Lemyze et al. [17]	Unknown	Retrospective observational study	1	Low	Early removal of a neck stabilisation can increase harm to patients after hanging due to raised intracranial pressure
Lin et al. [18]	Asia	Retrospective observational study	8633	High	Incidence of cervical spinal injuries in the urban area lightweight motorcyclist is very low. Pre-hospital protocol for application of a cervical collar brace to people who have sustained a lightweight motorcycle accident in the urban area should be revised to avoid unnecessary restraint and possible complications
Barkana et al. [13]	Israel	Retrospective observational study	36	Moderate	Life-threatening complications due to penetrating neck injury are common and may be overlooked if the neck is covered by a stabilisation device

### Risk of bias

The risk of bias assessment was performed using the ROBINS-I tool, which evaluates the following seven domains: (1) Bias due to confounding; (2): Bias in selection of participants; (3) Bias in classification of interventions; (4) Bias due to deviations from intended intervention; (5) Bias due to missing data; (6) Bias in measurement of outcomes; and (7) Bias in selection of the reported results, based on the presence or absence of some characteristic in “Low Risk”, “moderate risk”, “serious Risk”, “critical Risk” and “no information”. The quality of the evidence was used on the GRADE scale as in Table 2.

### Systematic review

As shown in Table 3, three studies out of the nine were undecided if spinal collars should be used in the pre-hospital setting with trauma patients. Underbrink et al. [21] showed that there was no difference when using a spinal collar. The presence of neurological deficit 6.5% vs. 5.3%, *p* = 0.69 was similar before and after protocol implementation in hospital mortality-adjusted Odd ratio = 0.56, 95% confidence interval 0.24–1.30, *p* = 0.18 which was similar to post-protocol implementation after adjusting for injury severity. Oosterwold et al. [19] stated spinal collars were used in 96.3% of trauma patients; however, 2.1% did not meet the criteria and 1.6% of the data was missing. 37.2% of patient’s spinal immobilisation was due to posterior midline spinal tenderness, where 5.7% were suspected as SI and 13.5% were due to painful distracting injuries. A total of 15.8% of patients were immobilised using non-standard methods due to side effects, including 0.9% who had worsening pain, 0.3% experienced shortness of breath, 0.6% due to combativeness or anxiety and 0.1% was due to worsening pain when supine.

**Table 3:** Summary of conclusions. Should spinal collars be used?

Study	Yes	No	Undecided	Notes
Underbrink et al. [21]			X	No differences in neurologic deficit
Oosterwold et al. [19]			X	Adverse effects of spinal immobilisation were incompletely documented
Vanderlan et al. [22]		X		Increased risk of death
Haut et al. [16]		X		Higher mortality in penetrating trauma and should not be used in every trauma patient
Brown et al. [14]			X	Unproven risk
Hauswalk et al. [15]		X		Immobilisation has little or no effect on neurologic outcome and can be deemed unnecessary
Lemyze et al. [17]		X		When a patient has hung a spinal collar can increase the intracerebral pressure, so not to use them at all or if they have been used them remove them as soon as possible
Lin et al. [18]		X		It needs to be revised to avoid unnecessary restraint and possible complications
Barkana et al. [13]		X		If spinal collars are using for penetrating injuries of the neck this may mean neck injuries are overlooked and covered by a device and new management guidelines concerning pre-hospital stabilisation are suggested

Kornhall et al. [6] found no reason to abandon the current guidelines but recommended triaging tools which would need further research. Brown et al. [14] also suggested that more research is needed as inconclusive results were found in their study. Table 3 also shows that 6 of the research papers concluded that spinal collars should not be used for trauma patients in the pre-hospital setting. Two papers reported that there was increased mortality associated with spinal collars. For example, Vanderlan et al. [22] demonstrated increased death associated with spinal collars; 35 patients died (Odds ratio 2.77, 95% CI 1.18–6.49, *p* < 0.02). In addition, Haut et al. [16] reported that unadjusted mortality was twice as high in the spinal immobilisation groups (14.7% vs. 7.2%, *p* < 0.001, Odds ratio of death for spine collar patients was 2.06). Furthermore, Hauswalk et al. [15] found reduced neurological disability in patients whose spine did not have spinal immobilisation. There was *a* < 2% chance that the spinal immobilisation has any benefit, in traumatic injury patients. This also suggests that better patient outcome is achieved when not using spinal collars. Lin et al. [18] and Barkana et al. [13] both suggested that spinal collars should not be used. Lin et al. [18] found that using collars on lightweight motorbike accidents was unnecessary and led to complications; only 63 out of 8633 had a cervical injury. The length of hospital stay was longer for patients who had spinal collars applied. Barkana et al. [13] suggest that spinal collars for traumatic neck injuries can hide an injury and may lead to it being missed. Penetrating neck injuries are likely to be associated with SI, the current guidelines need to be reviewed. In support of this, Lemyze et al. [17] reccomended that if a spinal collar was put on correctly by pre-hospital staff then this should still be removed as soon as possible. This is due to the risk of increased intracranial pressure for the patient. Ideally, spinal collars will never be applied by a pre-hospital clinician to a person who has experienced a strangulation trauma.

## Discussion

In this systematic review, key articles related to the use of spinal collars in the pre-hospital setting were synthesised qualitatively. The results demonstrate that there is a clear discussion that needs to be had and additional research that is needed to be undertaken to support this study. This is due to the findings showing that a majority of the studies (*n* = 6), disagree with spinal collars being used for pre-hospital trauma patients and the remaining three studies were either neutral or undecided. None of the studies were fully aligned with the current pre-hospital practice, and therefore, this needs to be reviewed and the confusion over this clinical element needs clarification. The main conclusion of our study is that the use of spinal collars is not supported by the literature but that there are only a handful of studies and a more extensive study needs to be performed to clarify the use of spinal collars in the pre-hospital setting.

### Common themes

Initially, to critically analyse the chosen literature, it is imperative to comprehend the common themes, to be able to endeavour to draw a concise and accurate conclusion. Primarily, a common theme that was identified is why the research study was undertaken and the aim of each of them. Underbrink et al. [21], Oosterwold et al. [19] and Kornhall et al. [6] were all acknowledged as having lack of evidence in the medical domain and the author’s impression that there is an element of uncertainty around the topic area. This is extremely central to the review and analysis because as an author the reason for this topic area was due to the confusion element. Clinicians have had no evidence-based material to give gold standard patient care and this needs to be sourced through literature reviews of current evidence and RCTs if deemed necessary.

Moreover, from the studies that have been deemed pertinent to this research, there is a common theme which is the type of study. The review of the current literature using data searching was one type of study [6, 19]. This is a vigorous part of research to compare the what papers are available to clinicians and see if from these a conclusion can be drawn or if it can be deemed that more research is needed. Oosterwold et al. [19], Haut et al. [16], Brown et al. [14], Hauswalk et al. [15], Lin et al. [18] and Barkana et al. [13] also shared the same theme of retrospective studies. These allow large volumes of data to be used, specifically picking elements which is important to the study using multivariate analyses. It is important to note that none of the studies found through our literature search were RCTs even though RCTs are deemed one of the most effective, accurate and reliable methods of gathering new data. This shows a lack of RCTs that are in the medical domain for clinicians to use in their evidence-based practice which provides an insight into why there is confusion around the subject area. Within the subject area of spinal collars, it may not be possible to undertake RCTs due to many reasons, including difficulties in obtaining ethical permission and altered patient care and outcomes without knowing the full effects. The patient may also be incapable of giving informed consent to participate and fully comprehend the potential risks involved with this type of study.

Subsequently, the preceding common theme established is the common conclusions that the 9 studies reached. Underbrink et al. [21] had no common theme with the other papers as it states that there is no significant difference when spinal collars were used. Conversely, Oosterwold et al. [19] and Kornhall et al. [6] did not seem to conclude on their research questions as set out, as no suppositions were drawn if spinal collars should be used in clinical practice. On the other hand, research has deemed that there is a lack of scoring and triage tool and assessments used in the pre-hospital setting. The authors have deemed that if these were in place and used to good effect this would give clinicians a better understanding of how to manage spinal injuries in trauma.

The most common theme throughout all of the selected research articles is that they conclude spinal collars should not be used in trauma patients [13, 15–18, 22], with only one study unable to draw a conclusion from their data [14].

### Bias

Research bias can be intentional, unintentional or both [20]. This can cause incorrect conclusions to be drawn by readers. Consequently, it is immoral and unethical to intentionally conduct biased research and also for this not to be looked for in another author’s research. A potential bias was found when critically analysing the research by Underbrink et al. [21]. This is due to the setting of the research which is based in the Rocky Mountain states of the USA. In this specific region, it is known that hospital trips can be longer and over harsher terrains than most other pre-hospital settings. This might mean that in this specific setting where the research is set, that an increasing number of interventions may be used to maintain the injury or injuries, which may not need to be carried out in more urban settings. Hence, this research could be biased towards the use of spinal collars, due to the terrain and distance, requiring intervention and managment on route to hospital. Furthermore, with the study being based in the USA, the research could be deemed biased due to the medical costs having to be paid by the patients. This could mean that there is a possibility that the treatment may be given or withheld from either the patient or clinician as opposed to other countries, such as, in the UK where all emergency treatment is at no cost to the patient.

Oosterwold et al. [19] can be deemed to have two biases present in their research. Initially, the research is only inclusive of blunt trauma injuries and is not inclusive of all trauma types. Therefore, this could bias towards certain age groups, for example, older people who are more likely to experience blunt trauma, as opposed to other types of trauma such as penetrating and lead to mistaken conclusions being drawn if to be applied to the all-inclusive population in practice. Similarly, as with the study by Underbrink et al. [21], the treatment may be biased and lead to incorrect conclusions as it is set in the USA where treatment is paid for. After extensive critical analysis there were no conclusive biases that were found which could have changed the conclusions and results in the studies by Kornhall et al. [6], Haut et al. [16], Brown et al. [14] and Hauswalk et al. [15]. Likewise, the study by Hauswalk et al. [15] was described to have no biases as it was undertaken by an independent clinician and was a blinded experiment.

Vanderlan et al. [22] were critically analysed to have biases present in their research. The biases found were that only one hospital was used in the research and within each hospital, there may be treatment preferences. One hospital may practically be in favour of spinal collars, therefore, the results that are produced could be biased and unable to change practice alone. The study by Lemyze et al. [17] could be deemed to be biased against using spinal collars. As only one patient was assessed, and the mechanism of hanging could mean that the patient suffered increased intracranial pressure which patients with other mechanisms of trauma may not experience. This research would have to be used with other research and other mechanisms to reduce the biases.

The study by Lin et al. [18] can also be deemed as biased as the setting was only an urban setting, where the mechanisms injury are likely to be at lower speeds, and therefore, less likely to cause SIs that may benefit from spinal collars. In addition, in urban areas, the conveyance time to hospital is likely to be short and consequently easier to be able to not use a spinal collar. However, when there is a long journey such as in rural areas over harsher terrain this may be more difficult to maintain, and this may mean a spinal collar would be used.

Lastly, Barkana et al. [13] can be deemed as biased in the way that the patient is selected. The patients were military casualties and likely to be young and fit patients who will anatomically and physically respond differently to other types of patients. For example, older patients or children will respond differently, therefore, this paper is not a representation of the whole population and would not be able to change civilian practice without additional evidences.

### Economic effects and implications for future practice or research

Oosterwold et al. [19] and Kornhall et al. [6] concluded that there are further economic effects for future practice and research required. Financial input is needed for future research as the studies state that there is a need for more extensive triaging tools for pre-hospital clinicians. This is primarily for the assistance to pre-hospital staff when deciding if and when to immobilise spinal trauma patients using spinal collars using triaging tools. This might be an expensive study, due to the large number of resources and patients that would be required to obtain valid and reliable evidence. The financial element needs to be considered when deciding if they should be completed and the benefit of it being undertaken.

Furthermore, Brown et al. [14], Lemyze et al. [17] and Lin et al. [18] would also require future research which would need economic factors considered. Brown et al. [14] stated that large prospective studies are needed to clarify the role of pre-hospital spinal immobilisation after torso gunshot wounds. Lemyze et al. [17] require more than one case to see if that spinal immobilisation does also increase the intracranial pressure and the effect this has on the patient. Lin et al. [18] will need future research in rural settings which may also require using more pre-hospital uses and pre-hospital distributors.

On the other hand, Underbrink et al. [21] would not have any future economic or other implication for future research. This is because the study stated that there is no difference between using spinal collars and not maintaining the patient spine. There is no further research that would be required in this study in the author’s opinion as a clear conclusion has been reached. Vanderlan et al. [22], Haut et al. [16], Hauswalk et al. [15] and Barkana et al. [13] also require no further research stated in their papers. This is due to all conclusive answer being achieved and where no spinal collars are recommended this would have a positive financial and economic impact. This is due to no spinal collars being required on pre-hospital ambulances which are a high-cost piece of equipment which have many different sizes that need to be available at all times.

## Why are cervical collars recommended or not?

Cervical collars are generally used to restrict movement whenever spinal motion restriction is indicated and its application is possible. The best form of spinal motion restriction uses a spine board, head blocks and immobilisation straps with and without a cervical collar [23]. However, evidence not only shows the benefits of spinal immobilisation but also reports adverse effects such as raised intracranial pressure, pain and discomfort, pressure ulcers, difficulties in airway management, restriction of respiration and dural sac compression [24–32]. This is especially true for the cervical spine which is particularly susceptible to secondary injury during transportation due to its inferior stability and accounts for > 29% of all spinal cord injuries [33, 34]. In another study which compared emergency immobilisation on neurological outcome over a 5-year period at two University hospitals, with comparable physician training and resources, less neurological disability was observed in immobilised patients, corresponding to *a* < 2% chance that immobilisation had any beneficial effects [15].

Despite these reservations, immediate immobilisation, especially in the case of cervical SIs, is a standard procedure performed by professional emergency care providers worldwide [3]. There is no agreement as to which immobilisation method is best to use and this is left to the discretion of the emergency services practitioner. However, a recent study compared the quality of spinal immobilisation using the vacuum mattress and spine board techniques [35]. The study found that cervical collars had no benefit in restricting movement using either the vacuum mattress or the spine board techniques, in agreement with other studies [36, 37]. Based on the general complications that accompany the use of spinal collars, and especially for cervical injuries, it remains questionable if the general application of cervical collars in every trauma patient is supported by the literature as best practice [3, 36, 37].

### Strength of evidence

The strength of evidence provided by these research papers is imperative to analysis, if papers can be used for challenging and revaluating/changing the way spinal collars are used in the pre-hospital setting in the future. The vigour of the evidence increases the validity of the conclusions drawn.

Through critical analysis, Underbrink et al. [21] and Kornhall et al. [6] have been deemed as strong evidence which can be used in further discussions on the topic area. It has been deemed as possessing strong elements, first, due to the large number of patients that have been included in these studies, allowing for the most reliable, valid and inclusive conclusions to be established. Furthermore, a conclusive answer has been drawn by the authors in this research using multiple outcome measures which make the conclusion more reliable. Lastly, Kornhall et al. [6] have been deemed as strong evidence and out of all the identified papers, it has the most robust evidence analysed. This paper has concluded through evidence-based results for specific potential spinal injuries, the time-critical threat to life patients, patients who are positive or negative to the NEXUS triage tool, recommendations for patients with an isolated penetrating injury and triaging tools based on clinical figures. Kornhall et al. [6] were able to address all of its aims such as over-triage, harm and costs with current management. With this paper having such extensive outcome measures, this increases the reliability, validity and the strength of the conclusions found.

Some of the studies [15, 16, 18, 22] are also deemed as strong evidence while some studies are based on a very large sample size in their research [16, 18, 22]. This has allowed for valid and reliable information to be recorded and lead to more convincing research and allowance for any anomalies. Hauswalk et al. [15] have used a smaller sample size but over 5 years, using two hospitals with two independent and blinded physicians which reduces the potential biases and limitations. On the other hand, the study by Oosterwold et al. [19] is regarded as weak evidence. Weak evidence is generally unreliable and invalid and consequently cannot be used on their own to help conclude and answer their question, requiring supplementary research to validate these studies. Additional research perceptibly has a financial and time element to consider. In addition, minimal number of papers were included in this study making the results less reliable and valid, from the papers that were included no combination studies were undertaken which can be vital in getting an accurate conclusive answer. Lastly, Oosterwold et al. [19] have also been deemed to be weak evidence due to the interpretation of “distracting injury” was lacking and adverse effects of spinal immobilisation were incompletely documented, therefore, not able to use this evidence to attempt to change current practice.

Other studies were also deemed as weak evidence since they failed to answer the pre-specified questions [13, 14, 17]. The results were predominantly based on what emergency surgical interventions were needed, which is a useful secondary outcome but does not answer the primary endpoint measurement. Lemyze et al. [17] only covered one case, and therefore, is weak evidence. This is a good indication of future research that needs to be completed but cannot be used for anything else in particular. Barkana et al. [13] were also analysed as weak evidence due to the low number of patients that were involved in the study. In addition, the patients studied in this paper were mainly young and extremely healthy males which is not representative of the general public and cannot be directly related to civilian life.

## Limitations

Within the selected research papers, some limitations need to be identified to assess the reliability and validity of each article. First, the main limitation in Underbrink et al. [21] is that it is not representative of an inclusive age bracket that has been undertaken in the study. The study has only included adults over the age of 60 years which means that not only did the study not include younger adults between 18 and 59 years of age but that this may have also excluded some types of trauma. The older adults are more exposed to types of trauma such as falls and blunt traumatic injuries as opposed to penetrating trauma such as stabbings and major road traffic accidents. Both the selective age group and also the type of trauma this paper is exposed to is not as inclusive of all elements of this subject area.

Penultimately, limitations in the study by Oosterwold et al. [19] could be deemed to be that it is only inclusive of blunt trauma injuries. Trauma covers a wide range of injuries that could all occur in the pre-hospital setting, requiring the use of spinal collars or different forms of immobilisation. To enable any significant change in practice, all trauma patients would need to be included to make sure all types of patients would benefit from any change in practice. Therefore, this paper on its own could not change the current practice but would be in line with other research covering other types of traumatic injuries. Vanderlan et al. [22], Haut et al. [16] and Barkana et al. [13] only focused on penetrating trauma which is a limitation as penetrating trauma is less than 50% of all trauma. Furthermore, Brown et al. [14] only included gunshot wound patients, so it is extremely limited in mechanism, and therefore, not inclusive of all trauma patients.

Kornhall et al. [6] have not stated any limitations, nonetheless through critical analysis a potential limitation could be that due to the country and setting of the research, there may be a cost element which is prioritised over the medical need of the patient. In the USA, for example, medical cover and insurance are worth a significant amount of money and that can determine when interventions are used. Regarding the use of spinal collars, either the patient, clinician or insurance personnel may have different feelings and intentions than purely for medical reasons. This must be taken into account when assessing the accuracy and validity of the paper.

The limitations of studies by Lemyze et al. [17] and Lin et al. [18] are due to a lack of patients. Lin et al. [18], only studied patients over 1 year, and therefore, not as large a set of data as needed to try and change current practice. Lemyze is a single-case study, so it is limited in the impact it can have without other research performed on a larger scale, to bring changes to current guidelines.

Within Hauswalk et al. [15], there were no obvious limitations found. A large cohort was used over two different hospitals where clinicians acted independently and were blinded to the study conditions.

## Conclusions

To conclude, from the papers that have been critically analysed it can be determined that the use of spinal collars is outdated and not essential in the pre-hospital use. Underbrink et al. [21] have been imperative in this finding and it has a large impact on the importance to the field of work. This is due to it being a large study concluding that there was no neurological deficit when using spinal collars in trauma patients. Therefore, this can be deemed to be an unnecessary intervention which has psychological and physical impacts along with side effects on the patient and their consequent recovery. Psychological effects include anxiety and combativeness and physical effects, such as increased pain when supine or in other areas and shortness of breath as unearthed in Oosterwold et al. [19].

However, at present, there is a distinct lack of evidence in being able to change clinical practice. The main way to do this will be to undertake RCTs. Even though they have negatives such as cost, the research shows the level of perplexity in this area and that RCTs are going to be the best way to navigate this area going forward.

From analysing the papers, an interesting element which has not been considered before is the importance of wholesome triaging of trauma patients and this can be done using appropriate tools. Oosterwold et al. [19] and Kornhall et al. [6] have deemed that patients have incorrectly had spinal interventions based on reduced triaging and if triaging had been better than the correct intervention would have been used. This is an element which could assist in assessing if spinal collars are better than not using them in pre-hospital, and therefore, should be considered in future research.

## Data Availability

All data are available within this manuscript.
